# Simultaneous Pancreas and Kidney Transplant Recipients From Pediatric Donors Have Comparable Outcomes to Those From Adult Donors, Regardless of the Type of Donation

**DOI:** 10.1097/TXD.0000000000001985

**Published:** 2026-07-20

**Authors:** Sandesh Parajuli, Camille Ylagan, Dave Patel, Paul E. Schindler, Sundeep Ram, Carrie Thiessen, David Aufhauser, Riccardo Tamburrini, Didier Mandelbrot, Dixon Kaufman, Jon Odorico

**Affiliations:** 1 Division of Nephrology, Department of Medicine, University of Wisconsin School of Medicine and Public Health, Madison, WI.; 2 Division of Transplantation, Department of Surgery, University of Wisconsin School of Medicine and Public Health, Madison, WI.; 3 UW Health Transplant Center, Madison, WI.

## Abstract

**Background.:**

Increased use of pediatric donors or donation after circulatory death (DCD) donors could enlarge the pancreas transplant donor pool. Pediatric donors and DCD donors are underutilized but are associated with similar outcomes following simultaneous pancreas and kidney (SPK) transplantation, compared with adult donation after brain death (DBD) donors. However, SPK outcomes from donors in whom both variables are present are unknown.

**Methods.:**

Recipients were categorized into 4 groups based on the age of the donor and types of donations: DBD/≥18 y, DBD/<18 y, DCD/≥18 y, and DCD/<18 y. Kidney and pancreas graft functions were compared at various time frames. Risk of graft failure was the primary outcome.

**Results.:**

Of 234 SPK recipients, 127 (54%) were DBD/≥18 y, 43 (18%) DBD/<18 y, 49 (21%) DCD/≥18 y, and 15 (6%) DCD/<18 y. There were no significant differences among the groups in overall or death-censored graft failure of either organ. Multivariable analysis (reference to DBD/≥18) showed no significant risk for pancreas uncensored graft failure for DBD/<18 y (hazard ratio [HR], 0.58; 95% confidence interval [CI], 0.23-1.44; *P* = 0.24), DCD/≥18 y (HR, 0.62; 95% CI, 0.26-1.48; *P* = 0.29), or DCD/<18 y (HR, 0.92; 95% CI, 0.31-2.79; *P* = 0.89). Pancreas graft function, as assessed by glycated hemoglobin, was comparable up to 5 y posttransplant or at the last follow-up. Kidney function, incidence of pancreas or kidney biopsy-proven rejection, posttransplant diabetes, length of stay, or readmission rates were not significantly different across the groups.

**Conclusions.:**

SPK transplants using DCD organs from pediatric donors can be used without compromising outcomes.

## INTRODUCTION

Simultaneous pancreas and kidney (SPK) transplantation is the treatment of choice for patients with insulin-dependent diabetes and advanced chronic kidney disease or end-stage kidney disease.^1^ Among patients with diabetes and kidney failure, SPK offers superior metabolic control, long-term insulin independence, mitigation of secondary diabetic complications, freedom from dialysis, a better quality of life, and longer life expectancy.^2-4^ It is estimated that between 1987 and 2012, 79 187 life-years (4.6 life-years per recipient) were “saved” by SPK transplantation in the United States.^5^ Despite advances in the medical and perioperative care of the SPK recipient, as well as careful recipient and donor selection, improved surgical techniques, and refined immunosuppressive protocols leading to better graft survival, there has been a decrease in the overall rate of pancreas transplantation in the United States and worldwide.^6^ This decline is multifactorial, including improving diabetes care leading to delayed progression of nephropathy in recipients, low enthusiasm for pancreas transplantation among providers leading to low referral rates, an aging and fattening donor population, an accumulation of comorbidities in both donors and recipients, and many more.^7-9^ In 2021, within the Eurotransplant pancreas transplant allocation system, only 16% of available pancreases were transplanted.^9^ In the United States, only approximately 9% of all donors provide a pancreas graft that is transplanted, and of pancreata recovered with the intent for transplant, the nonuse rate is as high as 23%–29%.^10^

Consequently, for a center to achieve significant pancreas transplant volume in the current era where fewer “ideal” pancreas donors are available, it is necessary to explore using donors with characteristics beyond the typical “ideal” donor, such as pediatric or donation after circulatory death (DCD) donors. Among SPK recipients from pediatric donors, reported outcomes are excellent and comparable to those from adult donors.^11,12^ Despite good results, pancreata from pediatric donors are underutilized. For example, only approximately 20% of transplanted pancreata are from donors <18 y.^10^ In 2024, of 1162 pancreata transplanted in the United States, 690 (59%) donors were between 18 and 34 y, 176 (15%) between 11 and 17 y, 37 (3%) between 6 and 10 y, and 29 (2%) were 1–5 y.^13^

Another strategy to increase the donor pool includes the utilization of pancreata from DCD donors. Although DCD transplantation is associated with inherent challenges of prolonged warm ischemia time and overall reduced organ quality compared with donation after brain death (DBD) donors, multiple studies have reported comparable outcomes after pancreas transplantation between DCD and DBD donors.^14-17^ Yet, only 2%–5% of SPK transplants in the United States are derived from DCD donors, and this has not significantly increased in recent years.^16^ Given the reluctance across the pancreas transplant community to use both pediatric and DCD donors, we considered that combining these 2 strategies of DCD and pediatric donors could further increase the donor pool. Recently, Paessler et al^18^ reported similar outcomes among kidney-only recipients from pediatric deceased donors, comparing 350 DCD donors with 10 721 DBD donors. However, outcomes among SPK recipients based on the donor’s age group (pediatrics versus adults) and type of donor (DCD versus DBD) have not been reported. In this study, we share our experience among all SPK recipients categorized into 4 different groups based on the donor age and type of donation. We hypothesized that SPK transplants from combined DCD pediatric donors could have inferior outcomes compared with those from DBD and adult donors.

## MATERIALS AND METHODS

### Study Population and Design

This single-center study from the University of Wisconsin-Madison included all adult (≥18 y) SPK only transplant recipients performed between January 01, 2016, and April 30, 2024. Recipients were divided into 4 categories based on the brain death criteria of the donor and donors’ age at the time of the transplant: DBD/≥18 y, DBD/<18 y, DCD/≥18 y, and DCD/<18 y. We chose <18 y based on the Organ Procurement and Transplantation Network policy allocating organ priority and treating <18 y as a “pediatric” candidate for organ transplantation.^19^ We excluded multiorgan transplant recipients with pancreas (ie, simultaneous liver-pancreas, etc), pancreas after kidney and pancreas transplant alone recipients. Pancreas graft failure and kidney graft failure were primary outcomes of interest and included all causes of graft failure, including death. Secondary outcomes included pancreas and kidney biopsy-proven acute rejection and death-censored graft failure (DCGF). Furthermore, we assessed graft function as assessed by glycated hemoglobin (HbA1c) for pancreas and serum creatinine and estimated glomerular filtration rate (eGFR) for the kidney. Furthermore, in the subgroup analysis, we looked at the outcomes among recipients with type 2 diabetes (T2D). Recipients were followed until both grafts failed or until the end of data analysis in 10/2025.

Pancreas DCGF was defined based on the current United Network for Organ Sharing criteria for pancreas graft failure, which include removal of the pancreas graft, reregistration for a pancreas transplant, registration for an islet transplant after receiving a pancreas transplant, or an insulin requirement that is ≥0.5 U/kg/d for 90 consecutive days.^20^ Kidney DCGF was defined as the return to dialysis or kidney retransplantation. Kidney delayed graft function (DGF) was defined as a need for dialysis within the first 7 d of transplant. We defined posttransplant diabetes as any need for a blood sugar-lowering agent.

This study was approved by the University of Wisconsin Institutional Review Board (IRB protocol number: 2014-1072). This study followed the Declaration of Helsinki. The clinical and research activities being reported were consistent with the Principles of the Declaration of Istanbul as outlined in “The Declaration of Istanbul on Organ Trafficking and Transplant Tourism.” Because of the nature of this study, informed consent specific to this research was not obtained from patients.

### Simultaneous Pancreas and Kidney Transplant Procedure

All pancreas transplants were accomplished using enteric drainage, side-to-side duodenojejunostomy to the proximal jejunum without a Roux-en-Y, and systemic venous drainage to the proximal right common iliac vein or distal inferior vena cava. In most cases, the kidney was placed contralaterally to the left iliac vessels.

### DCD Donor Pancreas Graft Procurement From Pediatric or Adult Donors

There was essentially no major difference in how we accepted or declined DCD donors if they were from pediatric or adult donors. Our DCD criteria, whether recovered as super-rapid recovery donors or normothermic regional perfusion (NRP) donors, were donors that progress from agonal (systolic blood pressure <70 mm Hg or oxygen saturation <70%) to cross clamp/flush in the case of super-rapid recovery or to on circuit/pump in the case of NRP in ≤30–35 min. All donors received IV heparin before withdrawal of support. For pediatric donors, there were no specific donation or logistics considerations that were different from those for adult donors. Since pediatric donors were more tolerant of the cold ischemia time, we were willing to import pancreata from significant distances.^21,22^ We do, however, have anatomical considerations. Our chief anatomical considerations were: (1) donor and splenic artery size. Splenic artery size must be >2.5 mm in diameter, with management including gentle dilation of the vessel with a hemostat on the backtable to break the typical arterial spasm that occurs in small healthy vessels, and performing an interrupted anastomosis with 7-0 prolene. Pediatric donors of pancreata were generally >20–25 kg in weight. We do not consider donor weight to recipient weight ratio an important factor (ie, relevance related to very small pediatric donors), an issue specifically concerning pancreatic function. We believe these can function sufficiently in any otherwise eligible recipient, including for mildly obese recipients with type II diabetes. The major consideration was the kidney size; therefore, we preferred to transplant these organs into patients with less fat and muscle mass. The size of the kidneys was important to not underdose renal mass. In this case, it was sometimes desirable to receive the kidneys en bloc if they were smaller than ~ 7 cm, as donor weight to recipient weight ratios have been identified as important in this situation. Again, we deemed this more relevant for the renal mass rather than the pancreatic mass.^12,23-25^

### Immunosuppressive Protocols

Our center-specific induction immunosuppression therapy was consistent throughout the study period; either a depleting agent (alemtuzumab or anti-thymocyte globulin) or a nondepleting agent (basiliximab) was used based on perceived immunological risk profile. Triple immunosuppression with tacrolimus, mycophenolic acid, and a prednisone taper was standard for all recipients. A few patients had early steroid withdrawal based on the immunological risk and patient request, as previously described.^26^

### Biopsy and Rejection Protocols

The 2 most common indications for kidney biopsy were an unexplained rise in serum creatinine and proteinuria, or a HLA donor-specific antibody-based protocol biopsy, as described before.^27^ Similarly, the common indications for pancreas biopsy were an unexplained rise in pancreatic enzymes, development of de novo donor-specific antibody, and unexplained hyperglycemia. If possible, we attempt to perform both graft biopsies in the setting of dual graft dysfunction among SPK recipients.^28^

The management of rejection was based on the severity and proximity of the transplant to the diagnosis of rejection, as described before.^27,28^ Briefly, kidney T-cell–mediated rejection was treated with a steroid pulse with or without anti-thymocyte globulin. Antibody-mediated rejection was treated with a steroid pulse, IVIG, with or without rituximab, with or without plasmapheresis. Treatment of pancreas rejection was based on the type and severity of rejection and was graded by the Banff criteria. T-cell–mediated rejection was treated with an IV steroid pulse with or without anti-thymoglobulin 6–12 mg/kg in 4–10 divided doses, while mixed rejection was treated with steroids, anti-thymoglobulin, IVIG, and plasmapheresis. Early antibody-mediated rejection was treated with steroids, IVIG, and plasmapheresis.

## STATISTICAL ANALYSIS

Continuous data were compared using Student *t* test or the Wilcoxon rank-sum test, as appropriate, whereas categorical data were analyzed using Fisher exact test or chi-square test. *P* values of ≤0.05 were considered statistically significant. Outcomes of interest were also analyzed as univariate and multivariate logistic regression models. DBD/≥18 y were considered as a reference. All variables from the baseline characteristics were included in the univariate analysis, and only variables with *P* < 0.05 in the univariate analysis were included in the multivariate analysis. Types of donors and donor age categories were included in the multivariate analysis regardless of the *P*-value. Multiple outcomes of interest were also presented as a Kaplan-Meier survival analysis, along with a subgroup analysis among T2D recipients. Further subgroup analysis was performed, categorizing recipients from donor age <12 y and body weight <30 kg. All analyses were performed using the MedCalc Statistical Software, Version 16.4.3 (MedCalc Software, Ostend, Belgium; https://www.medcalc.org; 2016).

## RESULTS

A total of 234 SPK recipients were transplanted during the study period and were analyzed, comprising 127 (54%) who were DBD/≥18 y, 43 (18%) who were DBD/<18 y, 49 (21%) who were DCD/≥18 y, and 15 (6%) who were DCD/<18 y. There were some differences in the baseline characteristics (Table 1) across the groups, mainly including the recipients’ sex, HLA mismatches, proportion of recipients with calculated panel reactive antibody >20%, proportion of male donors, and the donor’s body mass index (BMI). As expected, there were differences in the ages of the donors.

**TABLE 1.** Baseline characteristics

Donor age category (y)	Donation after brain death		Donation after circulatory death		*P*
	≥18 (n = 127)	<18 (n = 43)	≥18 (n = 49)	<18 (n = 15)	
Recipient: Male (%)	85 (67)	28 (65)	39 (80)	6 (40)	**0.04**
Recipient: White (%)	96 (76)	30 (70)	37 (76)	8 (53)	0.29
Mean recipient: age at transplant (y ± SD)	46.2 ± 10.0	48.0 ± 8.9	49.6 ± 9.2	41.3 ± 11.2	0.41
Type of diabetes					0.68
Diabetes I	74 (58)	28 (65)	26 (53)	8 (53)	
Diabetes II/other/unknown	53 (42)	15 (35)	23 (47)	7 (47)	
Preemptive transplant (%)	26 (21)	3 (7)	9 (18)	3 (20)	0.24
Previous transplant recipients (%)	4 (3)	3 (7)	2 (4)	0	0.59
Recipients BMI (kg/m^2^ ± SD)	27.1 ± 3.7	27.1 ± 3.4	27.2 ± 3.3	25.8 ± 3.9	0.69
Mean kidney donor profile index %	21.2 ± 17.4	26.9 ± 20.3	25.8 ± 14.5	36.5 ± 20.6	0.051
Mean HLA mismatch of 6 (±SD)	4.5 ± 1.26	4.5 ± 1.16	4.7 ± 1.0	4.8 ± 0.67	**0.01**
cPRA >20% (%)	15 (12)	9 (21)	1 (2)	3 (20)	**0.03**
Kidney cold ischemia time (h ± SD)	13.9 ± 4.2	13.9 ± 4.0	14.4 ± 3.9	17.1 ± 2.8	0.14
Pancreas cold ischemia time (h ± SD)	11.8 ± 4.0	11.8 ± 3.7	12.2 ± 3.7	14.7 ± 2.7	0.17
Induction agent (%)					0.62
Basiliximab	10 (8)	5 (12)	2 (4)	0	
Anti-thymocyte globulin	55 (43)	21 (49)	21 (43)	8 (53)	
Alemtuzumab	62 (49)	17 (40)	26 (53)	7 (47)	
Donor male (%)	77 (61)	23 (54)	40 (82)	13 (87)	**0.005**
Donor White (%)	109 (86)	31 (72)	40 (82)	12 (80)	0.24
Mean donor age (y ± SD)	30.3 ± 8.9	10.8 ± 4.6	28.3 ± 7.5	12.5 ± 4.8	**<0.001**
Mean donors BMI (kg/m^2^ ± SD)	23.7 ± 3.6	20.5 ± 3.7	23.4 ± 2.8	22.0 ± 5.3	**0.002**
Mean recipient minus (–) donor BMI (kg/m^2^ ± SD)	3.37 ± 5.0	6.7 ± 4.8	4.3 ± 5.7	3.8 ± 5.8	**0.005**
Donor cause of death: anoxia (%)	57 (45)	19 (44)	24 (49)	7 (47)	0.96
Import from the outside OPO (%)	58 (46)	24 (56)	27 (55)	11 (73)	0.16
Median distance between the transplant center and the donor center (miles, IQR)	101 (38–249)	112 (0–589)	107 (62–290)	313 (102–509)	0.34
Mean pancreas donor risk index	1.36 ± 0.45	1.29 ± 0.32	1.82 ± 0.45	1.93 ± 0.65	**<0.001**
Early steroid withdrawal (%)	20 (16)	5 (12)	3 (6)	1 (7)	0.31
Mean posttransplant hospital stay (d ± SD)	10.4 ± 10.9	8.6 ± 5.0	11.8 ± 7.9	10.3 ± 5.7	0.46
Kidney delayed graft function (%)	11 (9)	4 (9)	18 (37)	3 (20)	**<0.001**
Readmission within 30 d after transplant (%)	65 (51)	13 (30)	20 (41)	7 (47)	0.11

Bold signifies statistical significant with *P* < 0.05. BMI, body mass index; cPRA, calculated panel reactive antibody; IQR, interquartile range; OPO, Organ Procurement Organization.

The rate of kidney DGF was 9% in DBD/≥18 y and DBD/<18 y, while it was significantly higher at 37% in the DCD/≥18 y, and 20% among DCD/<18 y recipients (*P* < 0.001). The mean length of stay after the index transplant or readmission within 30 d of initial discharge was not different among the 4 groups.

**FIGURE 1.**
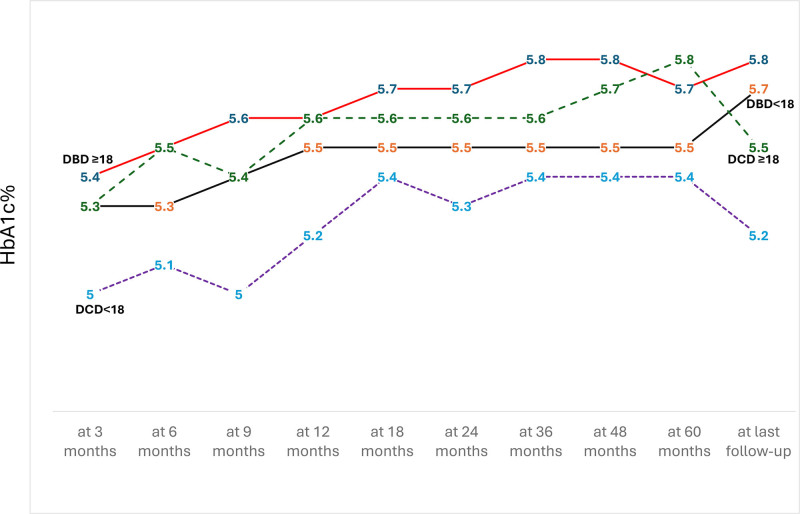
HbA1c trend of 4 different groups, at various times posttransplant, among those with a surviving pancreas graft. Comparing DBD/≥18 y with DCD/18 y, there was a statistically significantly lower HbA1c at 3 mo (*P* = 0.02), 6 mo (*P* = 0.01), and 9 mo (*P* = 0.03) posttransplant. DBD, donation after brain death; DCD, donation after circulatory death; HbA1c, glycated hemoglobin.

Assessing graft function, among those with surviving grafts (Table 2), mean HbA1c at various posttransplant intervals was comparable across the groups (Figure 1). Notably, however, patients receiving DCD and <18 y old donor pancreata experienced the lowest HbA1c levels.

**TABLE 2.** Graft and patient outcomes

Outcomes	Donor age category (y)	DBD		DCD		*P*
		≥18	<18	≥18	<18	
Mean HbA1c (%)	At 3 mo	5.4 ± 0.6	5.3 ± 0.7	5.3 ± 0.7	5.0 ± 0.5	0.92
	At 6 mo	5.5 ± 0.6	5.3 ± 0.7	5.5 ± 0.4	5.1 ± 0.5	0.33
	At 9 mo	5.6 ± 0.9	5.4 ± 0.6	5.4 ± 0.5	5.0 ± 0.4	**0.04**
	At 12 mo	5.6 ± 0.8	5.5 ± 0.7	5.6 ± 0.4	5.2 ± 0.4	0.14
	At 18 mo	5.7 ± 0.9	5.5 ± 0.7	5.6 ± 0.5	5.4 ± 0.4	0.65
	At 24 mo	5.7 ± 0.9	5.5 ± 0.7	5.6 ± 0.5	5.3 ± 0.3	0.34
	At 36 mo	5.8 ± 1.0	5.5 ± 0.5	5.6 ± 0.5	5.4 ± 0.4	0.053
	At 48 mo	5.8 ± 1.2	5.5 ± 0.9	5.7 ± 0.8	5.4 ± 0.3	0.68
	At 60 mo	5.7 ± 0.9	5.5 ± 0.6	5.8 ± 0.7	5.4 ± 0.4	0.59
	At last follow-up	5.8 ± 0.9	5.7 ± 0.9	5.5 ± 0.5	5.2 ± 0.4	0.24
Serum creatinine (mg/dL) and eGFR (mL/min)	At 3 mo	1.3 ± 0.4 (66 ± 21)	1.3 ± 0.3 (61 ± 15)	1.3 ± 0.3 (65 ± 16)	1.2 ± 0.4 (70 ± 19)	0.12 (0.051)
	At 6 mo	1.3 ± 0.4 (65 ± 21)	1.3 ± 0.3 (61 ± 15)	1.4 ± 0.5 (64 ± 18)	1.2 ± 0.3 (69 ± 14)	0.31 **(0.009**)
	At 9 mo	1.4 ± 0.4 (62 ± 21)	1.3 ± 0.3 (65 ± 15)	1.3 ± 0.3 (64 ± 15)	1.2 ± 0.2 (67 ± 15)	**0.002 (0.02**)
	At 12 mo	1.3 ± 0.4 (64 ± 21)	1.3 ± 0.4 (66 ± 17)	1.3 ± 0.3 (64 ± 16	1.1 ± 0.3 (76 ± 22)	0.26 (0.09)
	At 18 mo	1.4 ± 0.6 (63 ± 21)	1.2 ± 0.3 (72 ± 20)	1.3 ± 0.3 (65 ± 18)	1.2 ± 0.2 (68 ± 14)	0.07 (0.45)
	At 24 mo	1.4 ± 0.6 (63 ± 20)	1.2 ± 0.3 (72 ± 20)	1.3 ± 0.3 (67 ± 20)	1.1 ± 0.2 (70 ± 15)	0.06 (0.51)
	At 36 mo	1.3 ± 0.5 (64 ± 19)	1.1 ± 0.2 (75 ± 18)	1.3 ± 0.5 (63 ± 17)	1.3 ± 0.4 (66 ± 21)	0.22 (0.60)
	At 48 mo	1.3 ± 0.5 (66 ± 22)	1.1 ± 0.2 (74 ± 18)	1.3 ± 0.3 (63 ± 20)	1.2 ± 0.3 (71 ± 20)	**0.04** (0.46)
	At 60 mo	1.4 ± 0.7 (63 ± 21)	1.1 ± 0.2 (76 ± 18)	1.2 ± 0.3 (70 ± 16	1.4 ± 0.9 (67 ± 23)	**0.02** (0.56)
	At last follow-up	1.4 ± 0.6 (66 ± 22)	1.2 ± 0.4 (73 ± 20)	1.2 ± 0.3 (69 ± 18)	1.1 ± 0.2 (76 ± 10)	**0.006 (0.05**)
Acute rejection (%) during study period	Mean interval from transplant to 1st kidney rejection (mo)	55.3 ± 32.7	66.7 ± 39.1	56.1 ± 29.9	50.8 ± 36.9	**0.049**
	Patients with kidney rejection (%)	24 (19)	7 (16)	6 (12)	4 (27)	0.57
	Mean interval from transplant to 1st pancreas rejection (mo)	55.8 ± 33.9	71.1 ± 35.3	53.6 ± 31.0	55.4 ± 35.7	0.62
	Patients with pancreas rejection (%)	18 (14)	3 (7)	5 (10)	1 (7)	0.54
Outcomes at last follow-up (%)	Pancreas graft failure within 30 d	5 (4)	0	3 (6)	0	0.36
	Kidney graft failure within 30 d	1 (1)	1 (2)	1 (2)	0	0.79
	Uncensored graft failure (pancreas)	25 (20)	6 (14)	11 (22)	4 (27)	0.66
	DCGF (pancreas)	14 (11)	3 (7)	8 (16)	1 (7)	0.49
	Uncensored graft failure (kidney)	22 (17)	6 (14)	7 (14)	4 (27)	0.67
	DCGF (kidney)	12 (9)	4 (9)	3 (6)	1 (7)	0.90
	On antidiabetic agent among graft survival (PTD)	24 (19)	4 (9)	6 (12)	1 (7)	0.47
	eGFR <30 mL/min; among kidney graft survival	8 (6)	1 (2)	0	0	0.30

Bold signifies statistical significant with *P* < 0.05. DBD, donation after brain death; DCD, donation after circulatory death; DCGF, death-censored graft failure; eGFR, estimated glomerular filtration rate; HbA1c, glycated haemoglobin; PTD, posttransplant diabetes.

In assessing kidney graft function, serum creatinine was comparable; however, there were some differences in the eGFRs (Table 2). The rate of rejection of either graft was also comparable. Likewise, pancreas or kidney graft failure within 30 d of transplant was comparable. Also, at the time of last follow-up, there were no differences in the rate of graft failure or DCGF of either graft, or the proportion of recipients on antidiabetic agents, or eGFR <30 mL/min/1.73 m^2^.

**FIGURE 2.**
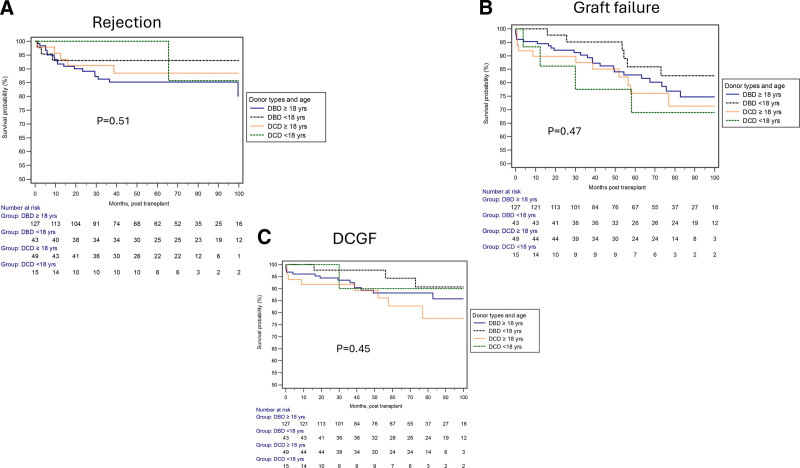
Pancreas outcomes: entire cohort. In the entire cohort, there was no significant difference in the rate of pancreas acute rejection (A; *P* = 0.54), pancreas graft failure (B; *P* = 0.47), or pancreas DCGF (C; *P* = 0.45). DBD, donation after brain death; DCD, donation after circulatory death; DCGF, death-censored graft failure.

Assessing the risk factors for pancreas graft failure, with reference to DBD/≥18 y, neither DBD/<18 y, nor DCD/≥18 y, nor DCD/<18 y were associated with an increased or decreased risk for pancreas graft failure (Table 3). The only factor associated with an increased risk for pancreas graft failure in multivariate analysis was kidney DGF (hazard ratio [HR], 2.86; 95% confidence interval [CI], 1.36-6.0; *P* = 0.005), whereas Caucasian recipient status was associated with a decreased risk of pancreas graft failure (HR, 0.48; 95% CI, 0.26-0.89; *P* = 0.02). This was further confirmed by the Kaplan-Meier survival analysis curve (Figure 2) for the various outcomes including rejection (Figure 2A), pancreas graft failure (Figure 2B), or pancreas DCGF (Figure 2C).

**TABLE 3.** Factors associated with pancreas graft failure

Covariate	Univariate analyses		Multivariate analyses	
	HR (95% CI)	*P*	HR (95% CI)	*P*
DBD, age ≥18 y	Ref	Ref	Ref	Ref
DBD, age <18	0.59 (0.24-1.46)	0.26	0.58 (0.23-1.44)	0.24
DCD, age ≥18 y	0.67 (0.57-2.37)	0.67	0.62 (0.26-1.48)	0.29
DCD, age <18	1.47 (0.51-4.23)	0.47	0.92 (0.31-2.79)	0.89
Recipient: male	1.10 (0.59-2.07)	0.75		
Recipient: White vs other	0.46 (0.25-0.84)	**0.01**	0.48 (0.26-0.89)	**0.02**
Age at transplant per y	0.99 (0.96-1.02)	0.62		
Type of diabetes: type 1 diabetes vs other	0.77 (0.43-1.38)	0.39		
Preemptive transplant	0.87 (0.40-1.87)	0.73		
Previous transplant	–	–		
Recipients’ BMI per kg/m^2^	1.02 (0.94-1.11)	0.67		
Kidney donor profile index per	1.01 (0.99-1.03)	0.17		
Mean HLA mismatch of per	1.09 (0.85-1.41)	0.49		
cPRA >20%	1.03 (0.44-2.42)	0.95		
Kidney cold ischemia time per h	1.02 (0.95-1.09)	0.66		
Pancreas cold ischemia time per h	1.03 (0.96-1.11)	0.40		
Nondepleting induction agent	1.57 (0.66-3.72)	0.30		
Donor male	0.72 (0.40-1.29)	0.27		
Donor White	0.73 (0.36-1.47)	0.38		
Donors’ BMI per kg/m^2^	1.02 (0.94-1.09)	0.67		
Recipient-donor BMI mismatch per kg/m^2^	0.99 (0.94-1.06)	0.93		
Pancreas donor risk index per 0.1	1.78 (1.09-2.89)	**0.02**	1.57 (0.87-2.83)	0.14
Import from outside OPO	1.04 (0.59-1.87)	0.88		
Early steroid withdrawal	0.79 (0.28-2.19)	0.64		
Kidney DGF	2.83 (1.49-5.39)	**0.002**	2.86 (1.36-6.0)	**0.005**

Bold signifies statistical significant with *P* < 0.05. BMI, body mass index; CI, confidence interval; cPRA, calculated panel reactive antibody; DBD, donation after brain death; DCD, donation after circulatory death; DGF, delayed graft function; HR, hazard ratio; OPO, Organ Procurement Organization; Ref, reference.

**FIGURE 3.**
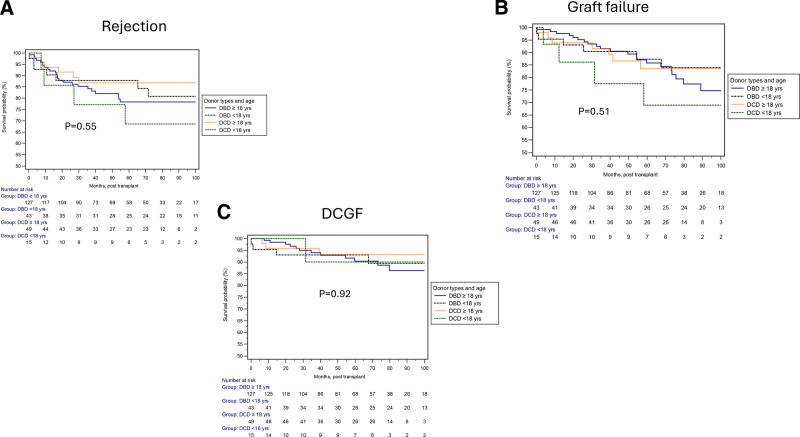
Kidney outcomes: entire cohort. In the entire cohort, there was no significant difference in the rate of kidney acute rejection (A; *P* = 0.55), kidney graft failure (B; *P* = 0.51), or kidney DCGF (C; *P* = 0.92). DBD, donation after brain death; DCD, donation after circulatory death; DCGF, death-censored graft failure.

Likewise, assessing the risk factors for kidney graft failure, with reference to DBD/≥18 y, neither DBD/<18 y, nor DCD/≥18 y, nor DCD/<18 y were associated with an increased or decreased risk for kidney graft failure (Table 4). The only factor associated with an increased risk for kidney graft failure in multivariate analysis was kidney DGF (HR, 2.84; 95% CI, 1.31-6.15; *P* = 0.008). Notably, higher recipient BMI was associated with a marginally decreased risk of kidney graft failure (HR, 0.90; 95% CI, 0.83-0.98; *P* = 0.03). This was further confirmed by the Kaplan-Meier survival analysis curve (Figure 3) for various outcomes, including rejection (Figure 3A), kidney graft failure (Figure 3B), or kidney DCGF (Figure 3C). Similar outcomes for acute rejection or DCGF of the pancreas or kidney were found by Cox regression analysis (data not shown).

**TABLE 4.** Factors associated with kidney graft failure

Covariate	Univariate analyses		Multivariate analyses	
	HR (95% CI)	*P*	HR (95% CI)	*P*
DBD, age ≥18 y	Ref	Ref	Ref	Ref
DBD, age <18	0.71 (0.28-1.75)	0.45	0.70 (0.28-1.75)	0.45
DCD, age ≥18 y	0.84 (0.36-1.95)	0.68	0.58 (0.23-1.44)	0.24
DCD, age <18	1.77 (0.61-5.13)	0.30	1.54 (0.53-4.49)	0.43
Recipient: male	0.76 (0.40-1.44)	0.40		
Recipient: White vs other	0.72 (0.36-1.42)	0.34		
Age at transplant per y	0.99 (0.97-1.03)	0.91		
Type of diabetes: type 1 diabetes vs other	0.84 (0.44-1.58)	0.58		
Preemptive transplant	0.62 (0.24-1.59)	0.32		
Previous transplant	0.55 (0.08-4.08)	0.56		
Recipients’ BMI per kg/m^2^	0.90 (0.82-0.98)	**0.01**	0.90 (0.83-0.98)	**0.03**
Kidney donor profile index per	1.01 (0.99-1.03)	0.18		
Mean HLA mismatch of per	1.06 (0.81-1.39)	0.68		
cPRA >20%	1.51 (0.67-3.43)	0.32		
Kidney cold ischemia time per h	1.03 (0.95-1.11)	0.46		
Pancreas cold ischemia time per h	1.05 (0.97-1.14)	0.23		
Nondepleting induction agent	1.38 (0.54-3.56)	0.50		
Donor male	1.03 (0.53-1.98)	0.94		
Donor White	0.96 (0.42-2.17)	0.92		
Donors’ BMI per kg/m^2^	1.01 (0.93-1.10)	0.75		
Recipient-donor BMI mismatch per kg/m^2^	0.96 (0.90-1.02)	0.25		
Pancreas donor risk index per 0.1	1.53 (0.89-2.66)	0.12		
Import from outside OPO	1.38 (0.73-2.61)	0.31		
Early steroid withdrawal	0.91 (0.32-2.56)	0.86		
Kidney DGF	2.48 (1.21-5.11)	**0.01**	2.84 (1.31-6.15)	**0.008**

Bold signifies statistical significant with *P* < 0.05. BMI, body mass index; CI, confidence interval; cPRA, calculated panel reactive antibody; DBD, donation after brain death; DCD, donation after circulatory death; DGF, delayed graft function; HR, hazard ratio; OPO, Organ Procurement Organization; Ref, reference.

**FIGURE 4.**
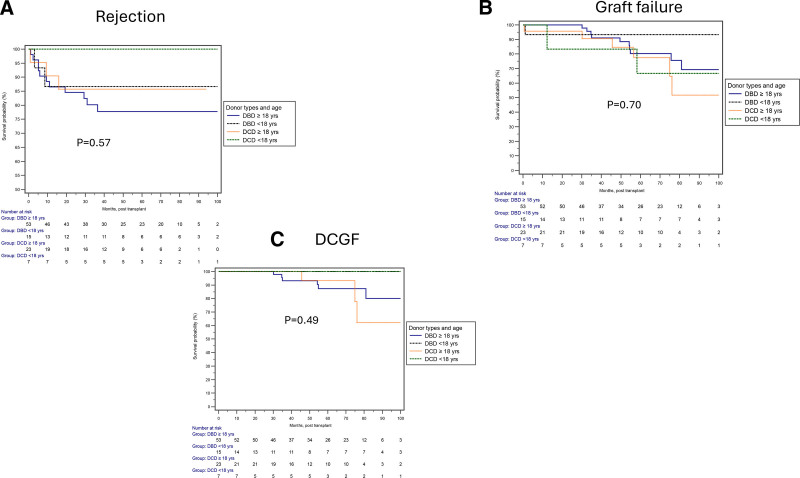
Pancreas outcomes: T2D. In a subgroup of recipients with type 2 diabetes (T2D), there was no significant difference in the rate of pancreas acute rejection (A; *P* = 0.57), pancreas graft failure (B; *P* = 0.70), or pancreas DCGF (C; *P* = 0.49). DBD, donation after brain death; DCD, donation after circulatory death; DCGF, death-censored graft failure.

**FIGURE 5.**
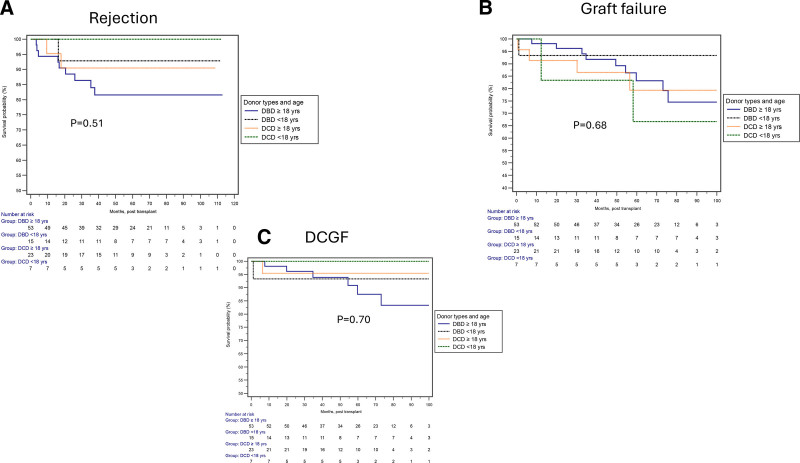
Kidney outcomes: T2D. In a subgroup of recipients with type 2 diabetes (T2D), there was no significant difference in the rate of kidney acute rejection (A; *P* = 0.51), kidney graft failure (B; *P* = 0.68), or kidney DCGF (C; *P* = 0.70). DBD, donation after brain death; DCD, donation after circulatory death; DCGF, death-censored graft failure.

A total of 98 SPK recipients (41.9%) had T2D. When outcomes were evaluated in this subgroup by Kaplan-Meier survival estimates, neither the age of the donor, nor the donation type, nor combined donor factors were associated with pancreas rejection, graft failure, or DCGF (Figure 4). Moreover, neither donor variable was associated with kidney rejection, graft failure, or DCGF (Figure 5).

The details of 15 recipients who received organs from DCD/<18 y donors are summarized in Table 5. The age of the donor ranged from 4 to 17 y, with weight between 17.6 and 85 kg. There was no technical pancreas graft failure in this group of recipients. Nor were there any pancreas graft failures within 30 d of the DBD/<18 y donor or recipients from a donor age <12 and weight <30 kg. There was a total of 8 pancreas graft failures within 30 d posttransplant in the ≥18 donor groups (5 in DBD/≥18 and 3 in DCD/≥18); of these, 2 were because of death with a functioning graft, 5 had thrombosis of the graft, and 1 had bowel perforation with impending breakdown of the duodenojejunostomy enteric anastomosis.

**TABLE 5.** Donor’s baseline characteristics and recipient’s BMI of DCD/<18 y

Year of donation	Donor age (y)	Donor height (cm)	Donor weight (kg)	Donor BMI (kg/m^2^)	Recipient height (cm)	Recipient weight (kg)	Recipient BMI (kg/m^2^)	NRP	Interval from transplant to the last follow-up (mo)	Pancreas graft failure at last follow-up	Cause of pancreas graft failure
2016	17	157.5	75.4	30.4	158	67.6	27.1	No	112.5	No	
2016	9	127	47	29.1	171	72.9	24.9	No	10.8	No	
2016	17	180.3	73.5	22.6	171	95.9	32.7	No	105.1	No	
2017	15	175.3	60	19.5	170.2	49.9	17.2	No	74.2	No	
2018	4	109.2	21.5	18.0	165	64	23.5	No	30	Yes	Chronic rejection
2018	15	154.9	50	20.8	188	106.7	30.1	No	88.8	No	
2018	16	175.3	85	27.6	147.5	46.5	21.3	No	3.7	Yes	Death with functioning graft
2018	4	107	17.6	15.3	157.5	59.4	23.9	No	58.2	Yes	Death with functioning graft
2019	12	145	33.8	16.0	156	60.6	24.9	No	77.2	No	
2019	5	122	24.5	16.4	161	72.3	27.8	No	72.2	No	
2020	16	180	84.1	25.9	176	85.3	27.5	No	61.6	No	
2020	16	171	70.5	24.1	177.8	94.6	29.9	No	58.2	No	
2021	16	170	80.8	27.9	162.6	76.8	29.0	Yes	12.3	Yes	Death with functioning graft
2021	11	140	40	20.4	154.9	57.4	23.9	No	14.6	No	
2024	15	142	31.1	15.4	178	73.9	23.3	No	16.1	No	

BMI, body mass index; DCD, donation after circulatory death; NRP, normothermic regional perfusion.

Until 2021, NRP was not used at our center. In this cohort, NRP was used in 4 cases: 1 in the DCD/<18 group and 3 in the DCD/≥18 group. Among these, the single recipient in the DCD/<18 group experienced pancreas graft failure because of death, while the remaining 3 recipients in the DCD/≥18 group had functioning kidney and pancreas grafts at last follow-up.

### Subgroup Analysis of Outcomes Among SPK Recipients From Donor age <12 y and Weight <30 kg

A total of 17 SPK recipients were from the donor age <12 y and body weight <30 kg (<age12/wt30) group. Of these, 3 were DCD and the remaining 14 were DBD. None of these recipients had early pancreas graft failure within 30 d posttransplant, while 4 had pancreas graft failure at last follow-up, 2 each because of death with a functioning graft and chronic rejection. Similarly, 1 recipient had early kidney graft failure within 30 d posttransplant because of graft thrombosis in the DBD/<18 y group. At last follow-up, 5 had kidney graft failure: 2 because of chronic rejection, 2 because of death with a functioning graft, and 1 with a torsed kidney. In the Cox regression analysis, with reference to age >12 y/wt >30 kg; in univariate analysis, the risk of pancreas graft failure was HR (1.11; 95% CI, 0.40-3.08; *P* = 0.85) and kidney graft failure was HR (1.98; 95% CI, 0.77-5.05; *P* = 0.16).

## DISCUSSION

In this large cohort of 234 SPK recipients, we report that SPK transplants using DCD organs from pediatric donors have similar posttransplant outcomes compared with either DBD or DCD adult donors. As “ideal” organ donor scarcity is a significant concern in recent years, using more combined DCD and pediatric donors could expand the donor pool. These data may encourage increased utilization of combined DCD/pediatric donors. Although limited by small sample size, there were no pancreas graft thromboses because of technical issues in pediatrics donor age <18 or even age <12 y.

In recent years, because of the growing gap between the number of organs available for transplantation and patients on waiting lists demand additional donor sources. Transplanting organs from DCD donors, formerly known as nonheart breathing (NHB) donors, is not a novel concept. Even in 1980, at the University Hospital Maastricht, an NHB-donor program was implemented after an in situ perfusion technique.^29^ Even then, among kidney transplant recipients, the long-term outcomes up to 5 y posttransplant were similar between NHB and heart-beating donors.^29^ In recent years, there has been a continuous increase in the number of organs recovered and successfully transplanted from DCD donors. In 1993, only 112 organs were recovered (81 of which were transplanted) from DCD donors, whereas by 2021, >10 000 organs (7333 of which were transplanted) had been recovered from DCD donors.^30^ In 2021, >20% of all recovered organs were from DCD donors.^30^ Despite some earlier challenges, various studies have reported comparable short- and medium-term outcomes among recipients from DCD or DBD donors in various solid organ transplantations, including heart, lungs, liver, kidney, pancreas, and islets.^16,31-35^ Another strategy to expand the donor pool would be the use of organs from pediatric donors. However, recently analyzing Organ Procurement and Transplantation Network data from 1994 to 2024, Nakagawa^36^ report pediatric donors (age <18 y) have remained relatively stable >29 y, with a trend toward fewer total organ donors in the past 14 y. In 2013, 10.6% of the deceased donors were <18 y, while in 2023 it was down to 5.4%.^37^ Outcomes of transplant recipients from pediatric donors are comparable to those of adult donors. In 1 recent study, from small pediatric donors aged <5 y, no significant differences were observed between pediatric and adult recipients regarding primary nonfunction, the incidence of proteinuria, vascular and urinary complications, or the cumulative incidence of acute rejection, even after receiving a single kidney from these small donors.^38^ Similar outcomes were reported by Schild et al^39^ in the past. Similarly, outcomes of pediatric pancreata for SPK transplants have been reported in multiple studies with similar outcomes as those of adult donors, although there were concerns about lower islet mass, fragile vascular anatomy, and increased risk of graft thrombosis.^12,40-42^ Even simultaneous en bloc kidney and pancreas transplantation from pediatric donors with excellent long-term glycemic control and kidney function to adult recipients has been reported.^23^ Combining these 2, here in this large cohort of 43 DBD pediatric donors and 15 DCD pediatric donors, we report similar posttransplant outcomes.

This study has the expected limitations of a single-center observational study, reflecting our specific population and clinical approach, which should be factored into the interpretation. Our expertise may have contributed to the favorable outcomes observed. We have made a concerted effort to clearly describe the selectivity of our approach, including considerations related to splenic artery diameter and its technical management, the rationale for our lack of concern regarding beta-cell mass, and our greater concern regarding renal mass. However, we are not suggesting changes to other center-specific donor selection criteria, nor are we advocating for the utilization of all pediatric DCD donors. Rather, our findings support the careful and selective use of appropriately chosen pediatric DCD donors within experienced transplant programs. Also, although we chose <18 y as pediatric donors based on common practices, some of these donors had adult-like physical statures based on their weight and BMI. Further limiting our analysis to donors <12 y of age and a weight <30 kg resulted in a very small sample size of only 17 recipients. However, this substantial data set with solid long-term granular data provides useful information for estimating risks and outcomes. Also, to the best of our knowledge, this study is the largest of its kind. In conclusion, although DCD organs from pediatric patients make up a small proportion of donors, the study demonstrates encouraging outcomes without evidence of a large signal of harm despite being underpowered to exclude moderate differences. These findings suggest that carefully selected pediatric DCD donors may represent an underutilized donor source for SPK transplantation at experienced centers.

## References

[1] Redfield RR Scalea JR Odorico JS. Simultaneous pancreas and kidney transplantation: current trends and future directions. Curr Opin Organ Transplant. 2015;20:94–102. 25565444 10.1097/MOT.0000000000000146 PMC4286161

[2] Ojo AO Meier-Kriesche HU Hanson JA. The impact of simultaneous pancreas-kidney transplantation on long-term patient survival. Transplantation. 2001;71:82–90. 11211201 10.1097/00007890-200101150-00014

[3] Sollinger HW Odorico JS Becker YT. One thousand simultaneous pancreas-kidney transplants at a single center with 22-year follow-up. Ann Surg. 2009;250:618–630. 19730242 10.1097/SLA.0b013e3181b76d2b

[4] Gruessner AC Gruessner RW. The current state of pancreas transplantation in the USA—a registry report. Curr Transplant Rep. 2018;5:304–314.

[5] Rana A Gruessner A Agopian VG. Survival benefit of solid-organ transplant in the United States. JAMA Surg. 2015;150:252–259. 25629390 10.1001/jamasurg.2014.2038

[6] Odorico JS Cooper M Dunn TB. Where have all the pancreas transplants gone and what needs to change? Curr Transpl Rep. 2019;6:285–293.

[7] Gruessner AC. A decade of pancreas transplantation—a registry report. Uro. 2023;3:132–150.

[8] Benjamens S Leemkuil M Margreiter C. A steady decline in pancreas transplantation rates. Pancreatology. 2019;19:31–38. 30448085 10.1016/j.pan.2018.11.003

[9] Miller G Ankerst DP Kattan MW. Pancreas transplantation outcome predictions-PTOP: a risk prediction tool for pancreas and pancreas-kidney transplants based on a European cohort. Transplant Direct. 2024;10:e1632. 38757051 10.1097/TXD.0000000000001632 PMC11098189

[10] Kandaswamy R Stock PG Miller JM. OPTN/SRTR 2023 annual data report: pancreas. Am J Transplant. 2025;25:S138–S192. 39947803 10.1016/j.ajt.2025.01.021 PMC12334190

[11] Fernandez LA Turgeon NA Odorico JS. Superior long-term results of simultaneous pancreas-kidney transplantation from pediatric donors. Am J Transplant. 2004;4:2093–2101. 15575914 10.1046/j.1600-6143.2004.00599.x

[12] Tamburrini R Odorico JS. Pediatric donors in pancreas transplantation: challenges and opportunities. Curr Opin Organ Transplant. 2025;30:304–314. 40539605 10.1097/MOT.0000000000001232

[13] Deceased donors recovered in the U.S. by donor age. Available at https://hrsa.unos.org/data/view-data-reports/national-data/#. Accessed January 24, 2026.

[14] Rathnasamy Muthusamy AS Friend PJ Dor FJ. DCD pancreas transplantation meta-analysis: ethical and technical considerations. Transplantation. 2017;101:e57. 27820782 10.1097/TP.0000000000001560

[15] Kopp WH Lam HD Schaapherder AFM. Pancreas transplantation with grafts from donors deceased after circulatory death: 5 years single-center experience. Transplantation. 2018;102:333–339. 28885491 10.1097/TP.0000000000001940

[16] Gruessner AC Saggi SJ Gruessner RW. Pancreas transplantation from donors after cardiac death—the US experience. Transplant Rep. 2022;7:100099.

[17] Fernandez LA Di Carlo A Odorico JS. Simultaneous pancreas-kidney transplantation from donation after cardiac death: successful long-term outcomes. Ann Surg. 2005;242:716–723. 16244546 10.1097/01.sla.0000186175.84788.50 PMC1409854

[18] Paessler A Brierley J Siebelink M. Long-term outcomes of pediatric kidney transplants from DCD and DBD donors: a comparative OPTN study. Transpl Int. 2025;38:14706. 41140298 10.3389/ti.2025.14706 PMC12548547

[19] Engen RM Perkins J Bartosh S. Pediatric priority in kidney allocation after the age of 18 years. Am J Transplant. 2024;24:850–856. 38272239 10.1016/j.ajt.2024.01.024

[20] Organ Procurement and Transplantation Network/United Network for Organ Sharing. Definition of pancreas graft failure. Pancreas Committee June 2015. Available at https://unos.org/wp-content/uploads/Definition-of-Pancreas-Graft-Failure.pdf. Accessed October 27, 2024.

[21] Kayler LK Lubetzky M Yu X. Influence of cold ischemia time in kidney transplants from small pediatric donors. Transplant Direct. 2017;3:e184. 28706987 10.1097/TXD.0000000000000668 PMC5498025

[22] Yagisawa T Kam I Chan L. Limitations of pediatric donor kidneys for transplantation. Clin Transplant. 1998;12:557–562. 9850450

[23] Tamburrini R Yang CY Philip JL. Simultaneous en bloc kidney and pancreas transplantation from pediatric donors: selection, surgical strategy, management, and outcomes. Am J Transplant. 2025;25:567–573. 39566660 10.1016/j.ajt.2024.11.016

[24] Al-Qaoud TM Odorico JS Al-Adra DP. Pancreas transplants from small donors: are the outcomes acceptable? A retrospective study. Transpl Int. 2020;33:1437–1446. 32749728 10.1111/tri.13711

[25] Tamburrini R Hidalgo S Leverson G. Importing pancreata for transplantation: a single-center experience across evolving allocation eras. Front Transplant. 2025;4:1698617. 41488372 10.3389/frtra.2025.1698617 PMC12756387

[26] Aziz F Parajuli S Kaufman D. Induction in pancreas transplantation: T-cell depletion versus IL-2 receptor blockade. Transplant Direct. 2022;8:e1402. 36505900 10.1097/TXD.0000000000001402 PMC9722744

[27] Parajuli S Joachim E Alagusundaramoorthy S. Subclinical antibody-mediated rejection after kidney transplantation: treatment outcomes. Transplantation. 2019;103:1722–1729. 30507740 10.1097/TP.0000000000002566

[28] Parajuli S Arpali E Astor BC. Concurrent biopsies of both grafts in recipients of simultaneous pancreas and kidney demonstrate high rates of discordance for rejection as well as discordance in type of rejection—a retrospective study. Transpl Int. 2018;31:32–37. 28672081 10.1111/tri.13007

[29] Daemen JW Kootstra G Wijnen RM. Nonheart-beating donors: the Maastricht experience. Clin Transpl. 1994;303:16. 7547551

[30] Barboza AB Dhanani NH Browning K. Trends in donation after circulatory determination of death donor utilization: lessons from Houston. Transplant Rep. 2023;8:100135.

[31] Jolliffe J Brookes J Williams M. Donation after circulatory death transplantation: a systematic review and meta-analysis of outcomes and methods of donation. Ann Cardiothorac Surg. 2025;14:11–27. 39944506 10.21037/acs-2024-dcd-0132 PMC11811570

[32] Abul Kashem M Loor G Hartwig M. A multicenter analysis of lung transplantation outcomes comparing donation after circulatory death and donation after brain death. JHLT Open. 2024;6:100132. 40145031 10.1016/j.jhlto.2024.100132 PMC11935477

[33] Ziogas IA Kakos CD Esagian SM. Liver transplant after donation from controlled circulatory death versus brain death: a UNOS database analysis and publication bias adjusted meta-analysis. Clin Transplant. 2022;36:e14521. 34689372 10.1111/ctr.14521

[34] Trotter PB Jochmans I Hulme W. Transplantation of kidneys from DCD and DBD donors who died after ligature asphyxiation: the UK experience. Am J Transplant. 2018;18:2739–2751. 29947090 10.1111/ajt.14989 PMC6221073

[35] Doppenberg JB Nijhoff MF Engelse MA. Clinical use of donation after circulatory death pancreas for islet transplantation. Am J Transplant. 2021;21:3077–3087. 33565712 10.1111/ajt.16533 PMC8518956

[36] Nakagawa T. 406.7: Pediatric organ donation and transplantation trends in the USA. Transplantation. 2025;109:S104–S104.

[37] Ajay K Israni D Alina M. OPTN/SRTR 2023 annual data report: deceased organ donation. Available at https://srtr.transplant.hrsa.gov/ADR/Chapter?name=DOD&year=2023#tab:DD-donor-summary. Accessed January 26, 2026. 10.1016/j.ajt.2025.01.026 PMC12334191 39947809

[38] Feng R Zhong B Liang H. Outcomes of single kidney transplantation from small pediatric donors aged <5 years: comparative analysis between pediatric and adult recipients. Ren Fail. 2025;47:2562446. 40968503 10.1080/0886022X.2025.2562446 PMC12451948

[39] Schild R Carvajal Abreu K Büscher A. Favorable outcome after single-kidney transplantation from small donors in children: a match-controlled CERTAIN registry Study. Transplantation. 2024;108:1793–1801. 38685197 10.1097/TP.0000000000004993

[40] Odorico JS Heisey DM Voss BJ. Donor factors affecting outcome after pancreas transplantation. Transplant Proc. 1998;30:276–277. 9532034 10.1016/s0041-1345(97)01263-3

[41] Spaggiari M Di Bella C Di Cocco P. Pancreas transplantation from pediatric donors: a single-center experience. Transplantation. 2018;102:1732–1739. 29620617 10.1097/TP.0000000000002208

[42] Choudhary D Rally S Panjathia A. Small donors, big impact: optimizing organ utilization in simultaneous pancreas and kidney transplantation from extra small pediatric donors. Clin Transplant. 2024;38:e15448. 39229679 10.1111/ctr.15448

